# Automated skin biopsy histopathological image annotation using multi-instance representation and learning

**DOI:** 10.1186/1755-8794-6-S3-S10

**Published:** 2013-11-11

**Authors:** Gang Zhang, Jian Yin, Ziping Li, Xiangyang Su, Guozheng Li, Honglai Zhang

**Affiliations:** 1 School of Information Science and Technology, SUN YAT-SEN University, Guangzhou, 510275, China; 2 The Second Affiliated Hospital of Guangzhou University of Chinese Medicine, 510120, China; 3 School of Automation, Guangdong University of Technology, Guangzhou, 510006, China; 4 The Third Affiliated Hospital of SUN YAT-SEN University, Guangzhou, 510630, China; 5 Department of Control Science and Engineering, Tongji University, Shanghai, 201804, China; 6 Guangzhou University of Chinese Medicine, Guangzhou, 510120, China

## Abstract

With digitisation and the development of computer-aided diagnosis, histopathological image analysis has attracted considerable interest in recent years. In this article, we address the problem of the automated annotation of skin biopsy images, a special type of histopathological image analysis. In contrast to previous well-studied methods in histopathology, we propose a novel annotation method based on a multi-instance learning framework. The proposed framework first represents each skin biopsy image as a multi-instance sample using a graph cutting method, decomposing the image to a set of visually disjoint regions. Then, we construct two classification models using multi-instance learning algorithms, among which one provides determinate results and the other calculates a posterior probability. We evaluate the proposed annotation framework using a real dataset containing 6691 skin biopsy images, with 15 properties as target annotation terms. The results indicate that the proposed method is effective and medically acceptable.

## Background

With the rapid development of computer-aided diagnosis, increasingly more digital data have been stored electronically. It has been a great challenge for doctors and experts to effectively analyse these data. Introducing the power of computational intelligence into this analysis problem would be meaningful and practical, with the potential not only to ease the burden of doctors but also to save time so that doctors and experts can pay more attention to confusing and difficult cases [1].

In skin disease diagnosis, histopathological data provide a microscopic view of skin tissue architecture, which contributes to the correct diagnosis of skin diseases. Microscopic analysis of skin tissue provides further information about what happens under the skin's surface. To confirm a skin disease, on the one hand, doctors should have a clear understanding of the patient's medical history and careful observations of the skin eruption. On the other hand, histopathological data are of great necessity. For example, different patients may appear to have the same rash; however, differences in their histopathological data can distinguish them and aid in diagnosis. Histopathological data provide a comprehensive view of the presence of disease and its effects on patients. Some skin diseases, especially benign skin tumours and skin cancer, should be diagnosed using histopathological information. The information we extract from the data can help a doctor judge a patient's condition, estimate the prognosis, direct treatment, and evaluate the curative effects of treatments. For undiagnosed disease, complete histopathological data can provide an initial assessment of a condition's nature and severity.

Generally, there are two levels of skin disease diagnosis: skin surface inspection [2] and skin biopsy image analysis [3]. The former is a diagnostic procedure that can roughly be reached after routine exams, including observation and the physical examination of skin lesions, whereas the latter is a complement of the former [4,5], utilised in cases where the doctor has less confidence or even cannot make a decision based only on an inspection of the skin surface. As indicated in histopathological studies, skin biopsy images reveal further information about what happens beneath the skin's surface at a microscopic level [4,6]. Therefore, the results of skin biopsy image analysis could be explained more accurately than observations of the surface. For a medically acceptable diagnosis, many skin biopsy image cases are usually required to identify the significant changes associated with that specific diagnosis and differentiate them from those of similar skin diseases [7]. Because understanding skin biopsy images requires more professional knowledge and richer experience [8] than inspecting the skin's surface, it becomes a great challenge for doctors to correctly interpret huge number of skin biopsy images.

**Figure 1:**
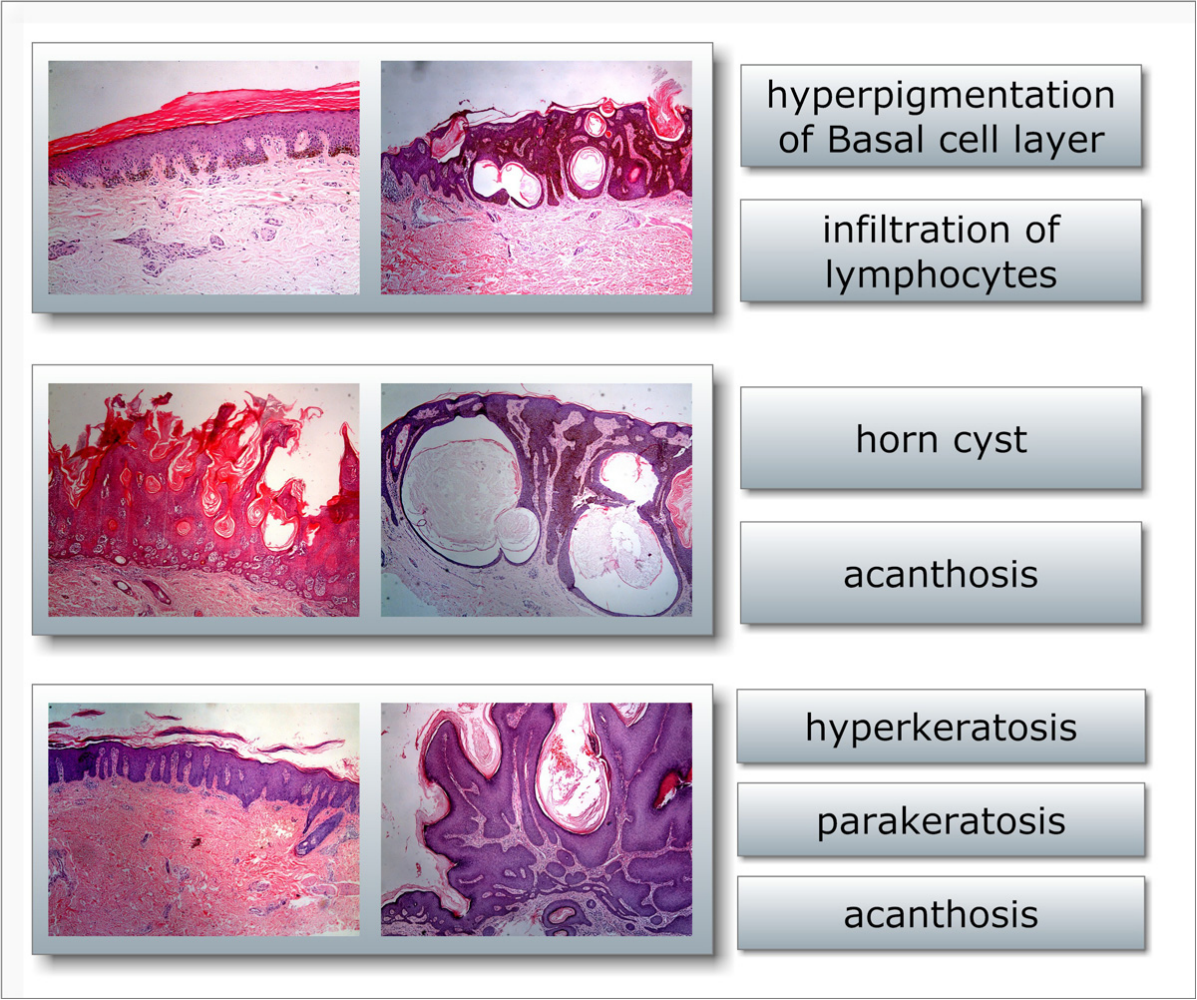
**Examples of skin biopsy image annotation**. An example of skin biopsy image annotation in the evaluation dataset. Each row has two images with the same annotation terms (the rightmost column). It can be observed that though annotated with the same terms, images in each row vary significantly in either colour, texture, local structure or other characteristics. These differences pose great challenges for automated annotation.

**Figure 2:**
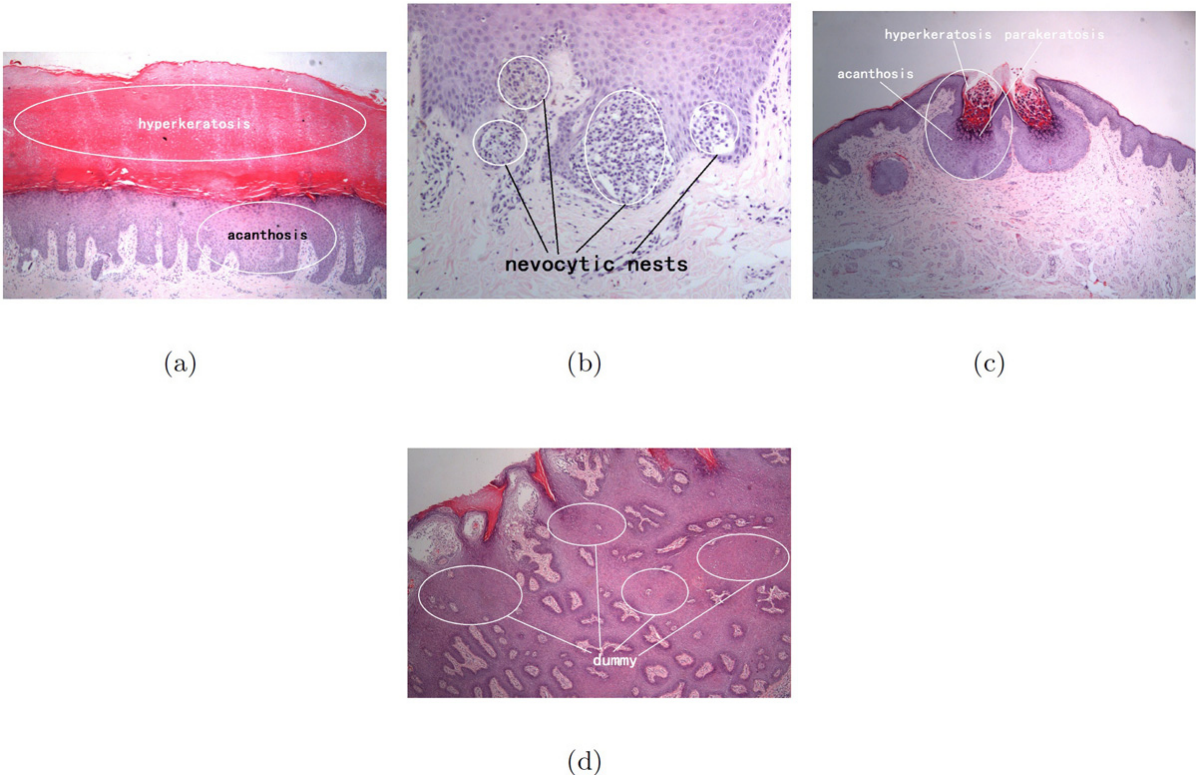
**Correspondence between annotation terms and local regions of an image**. Correspondence between annotation terms and regions within images. 4 subfigures show different types of correspondence. (a). 1 region to 1 term (b). *m *regions to 1 term (c). 1 region to *m *terms (d). a region does not correspond to any term.

An important step in skin biopsy image analysis is to annotate an image with a set of standard terms as a professional description of what is happening in the tissues. Due to the large number of biopsy images, computer-aided automated annotation methods have been investigated [1]. However, the task of automating skin biopsy image annotation poses at least two significant challenges. The first is the implicit ambiguity between annotation words and images. From clinical experience, a doctor can recognises skin biopsy images based on his expertise without explicitly attaching annotation terms to the exactly corresponding regions. What we can obtain is a whole image associated with a set of annotation terms, as indicated in Figure 1. The ambiguity also appears in the relationship between numbers of terms and corresponding regions. Figure 2 illustrates one-to-one, one-to-many, many-to-one and many-to-many relationships between between terms and regions. The second challenge is the complexity and variety of local regions annotated with the same term. The complexity and variety comprise differences in the texture, shape, size, color, magnification and even resolution of local regions, as shown in Figure 1. Two images in a row may share the same annotation terms but have totally different appearances. Hence, it is a great challenge to construct an automated annotation model that captures essential features for the terms.

Currently, several attempts to undertake the automated histopathological image analysis problem have been reported. Metin N. et al. [1] reviewed some important work on histopathological data analysis. They reviewed studies on different information source processing, segmentation and feature extraction methods for different application backgrounds and model training algorithms. Syed et al. [9] presented an analysis of feature extraction methods for bag-of-features representations of histopathological images. Juan C. Caicedo et al. [10] proposed a histopathological image classification method based on bag-of-features and a kernel-function-based model training algorithm. They approached the skin cancer histopathology image classification problem by representing images through bag-of-feature methods. However, they solved the problem as a traditional single instance learning problem [11] with a kernel machine. Though widely used in histopathological image feature extraction, bag-of-features don't, in fact, reveal the inner structures of histopathological images, and most important, it loses original information to some extent [12].

Much of the work in skin image recognition has been reported publicly. We review two important works closely related to our work here. Bunte et al. [13] proposed a novel machine learning method for skin surface image classification. They noticed that existing skin surface image feature extraction methods are only differently weighted strategies of color space. Hence, if an optimal weighted strategy is learned from the training dataset, it can achieve very good performance. In their work, an optimal weights vector is learned through a maximal margin classification algorithm, realising the idea that instead of finding a proper weighting, they derived one. However, their method is not suitable for our task. On the one hand, in their work, manual labelling of normal and lesion regions is required for each skin surface image. Because understanding a skin biopsy image requires more skill and expertise than understanding a skin surface image, this requirement would be a heavy burden for doctors. On the other hand, in the work of Bunte et al., only RGB colour space-based features are used, which cannot fully describe the essential features of biopsy images, e.g., texture, local structures and even visual edges. Moreover, biopsy images are often stained for clearer illustration of tissue structures and different types of cells, which would lead to the failure of purely colour-based feature extraction methods.

Another work that should be emphasised is on Drosophila gene image annotation, proposed by Li et al. [12]. They addressed the problem of the automated annotation of *Drosophila *embryogene expression patterns in a multi-instance multi-label learning (MIML) framework [14]. Annotation terms are associated with groups of images corresponding to different embryogene developmental stages, but more specifically, the terms are in fact associated with some patches within the group of images. They solve the problem by regarding each image group as a multi-instance sample and annotated terms as labels attached to the sample. They proposed two MIML algorithms for model training. To express a group of images as a bag, they adopt a block division method to generate equal-size patches as instances. Though the general framework of [12] is consistent with our task, it is not naturally suited to skin biopsy image annotation, as *Drosophila *embryogene images do not contain complex inner structures, textures or colours. Therefore, equal-size block division does not make sense for our task.

In this article, we propose a novel automated annotation framework based on the theory of multi-instance learning. Multi-instance learning is a special learning framework introduced by Dietterich et al. [15] to solve the drug activity prediction problem. Different from single-instance learning, samples in multi-instance learning (also called bags) are composed of several instances with potential concept labels, only the concept labels of bags are known. For binary classification tasks, a bag is positive if and only if it contains at least one positive instance and negative otherwise. The task of multi-instance learning is to predict the labels of unseen bags by training a model with labelled bags.

We first show that the skin biopsy image annotation task can naturally be decomposed into several binary multi-instance classification tasks. Then, by applying a graph-cutting algorithm and region-based feature extraction methods, we propose an effective method of expressing each skin biopsy image as a bag whose instances are regions. Finally, we propose two algorithms for model building. One is discriminative and produces a binary output indicating whether a given image should be annotated with a certain term. The other one models the conditional distribution *p*(*t_i_|I*, *D*) to calculate the posterior probability of annotating an image *I *with a term *ti*, given a training dataset *D*.

## Methods

In this section, we first show the intuition behind the proposed algorithm framework, then, following Gurcan et al.'s proposal[1], present the proposed algorithm framework as three steps:

1. Multi-instance sample representation

2. Feature extraction

3. Training of learning algorithms

**Figure 3:**
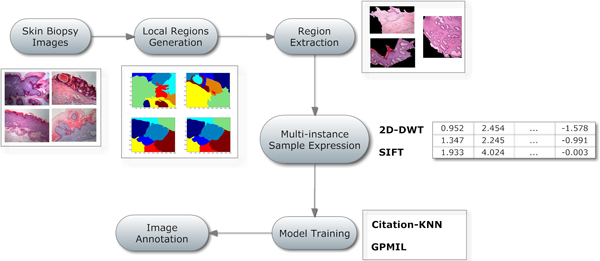
**Main framework**. An overview of the proposed skin biopsy image annotation framework. The input images are partitioned by a graph cutting algorithm through which local regions are generated. Feature extraction is applied to each generated local region to obtain a vectorial expression. Finally two multi-instance learning models are trained.

Figure 3 illustrates the framework of the above three steps. We should note that the proposed framework is adaptable and flexible because it only provides a general framework and different implementations can be replaced according to the application domain.

### Formulation

The proposed annotation framework is motivated by the nature of skin biopsy image recognition, which can be naturally expressed as a multi-instance learning problem. To make this intuition clearer, it is necessary to review the procedure of manually annotating skin biopsy images. From dermatopathological clinical experience, we can see that a set of standard terms are used by doctors to annotate an image. However, doctors are not required to explicitly record the correspondence between standard terms and regions within a given image, leading to the terms ambiguity described in the previous section. Because terms are actually associated with certain local regions, it is not reasonable to connect each region of an image to all associated terms, which results in poor models from a machine learning perspective [16]. As illustrated in Figures 2.(a)-(d), regions within a given image may have different relationships to the attached terms. It is time-consuming to manually label each region with a set of terms to meet the requirement of traditional single-instance learning. For this reason, by regarding each image as a bag and regions within the image as instances, multi-instance learning is naturally suitable for the annotation task. According to the basic assumption of multi-instance learning [15], a bag can be annotated with a term if it contains at least one region labelled with that term. Otherwise, the bag cannot be annotated with that term. Thus, we can build a set of binary multi-instance classifiers, each of which corresponds to a term. Given an image, each classifier outputs a Boolean value indicating whether its term should be annotated to the image. Thereby, we can address the term ambiguity within a multi-instance learning framework.

Another challenge is how to effectively represent an image as a multi-instance sample, or a bag. The key problem is how to partition an image into several regions to construct instances. Skin tissue is microscopically composed of several different structures, and a doctor needs to inspect them individually to determine abnormal areas. Regions of a skin biopsy image should be divided according to the structures of skin tissue to come up with a feature description for each part, but clustering-based algorithms [17] may not generate contiguous regions. Hence, we apply an image-cutting algorithm, namely Normalized Cut (NCut) [18], to generate visually disjoint local regions. Prior knowledge in dermatopathology suggests that on the one hand, examining an individual visually disjoint region is sufficient to annotate it in most cases, and on the other hand, there is not considerable relationship between terms to be annotated in a given image. The former supports the application of our image-cutting method, and the latter allows us to decompose the annotation task in to a set of multi-instance binary classification tasks.

Formally, let *D *= {(*I_i_*, *T_i_*)*|i *= 1, ..., *n*, *I_i _*∈ *I*, *T_i _*⊆*T*} be a set of skin biopsy images associated with a set of annotated terms, where *T *= {*t*_1_, *t*_2_, ..., *t_m_*} is a set of standard terms for annotation and *I *is a set of images. Each image is stored as a pixel matrix in 24k RGB colour space. The task is to learn a function *f *: *I → *2*^T ^*given *D*. When given an unseen image *I_x_*, *f *can output a subset of *T *corresponding to the annotation terms of the given image *I_x_*.

We first apply a cutting algorithm to generate visually disjoint regions for each image, given by *I_i _*= {*I_ij_|j *= 1, ..., *n_i_*}, where *n_i _*is the number of regions in image *I_i_*, followed by a feature extraction procedure to express each generated region as a feature vector. Then, we train the target model through two algorithms.

### Skin biopsy image representation

Now we present a method for representing a skin biopsy image. First, express each image as a bag of regions as instances, and then apply two transformation-invariant feature extraction methods to further express them as vectors.

#### Multi-instance sample representation

To generate visually disjoint regions, we adopt a famous graph-cutting algorithm, Normalized Cut (NCut), proposed by Shi et al. [18] in 2000, aimed at extracting perceptual groupings from a given image. In constract with clustering-based image segmentation algorithms, e.g., [17], NCut extracts the global impression of a given image, i.e., disjoint visual grouping. To make this article self-contained, we briefly present the main idea of NCut.

NCut approaches the segmentation of an image as a graph cutting problem. It constructs a local connection between neighbour pixels within an image. Vertices of the constructed graph are pixels, and the weights of edges are similarity between pixels. The problem of NCut is to find a cut that minimises in-segment similarity and maximises cross-segment similarity. Formally, supposing there is a graph *G *= (*V*, *E*), we aim to find an optimal cut that partitions it into two disjoint sets *A *and *B*, where *A *∩ *B *= *∅ *and *A *∪ *B *= *V*. A measure is defined in Eq. 1 as optimal graph cutting:

(1) $$N\;cut\left(A,B\right)=\frac{cut\left(A,B\right)}{assoc\left(A,V\right)}+\frac{cut\left(A,B\right)}{assoc\left(B,V\right)}$$

where $cut\left(A,B\right)=\sum_{u\in A,v\in B}w\left(u,v\right)$, *w*(*u, v*) is the weight of the edge between vertices *u *and *v*, and $assoc\left(A,V\right)=\sum_{u\in A,t\in \;V}w\left(u,t\right)$ is the summed weights of the edges between the vertices in segment *A *and any other vertices in graph *G*. Because graph *G *is locally connected, a binary column vector *x*_|*V*|×1 _can be defined to indicate whether a vertex belongs to subset *A*. The goal of NCut is to find a cut that minimises *Ncut*(*A*, *B*), as Eq. 2 shows.

(2) $$min_{x}N\;cut\left(x\right)$$

According to [18], the solution to Eq. 2 captures a visual segmentation of an image whose underlying idea is naturally consistent with the clinical experience of skin biopsy image recognition. Eq. 2 can be solved as a standard Rayleigh quotient [19]. We ignore the detailed procedure for brevity. The computational time complexity of NCut for a given image is *O*(*n*^2^), where *n *is the number of pixels in an image.

**Figure 4:**
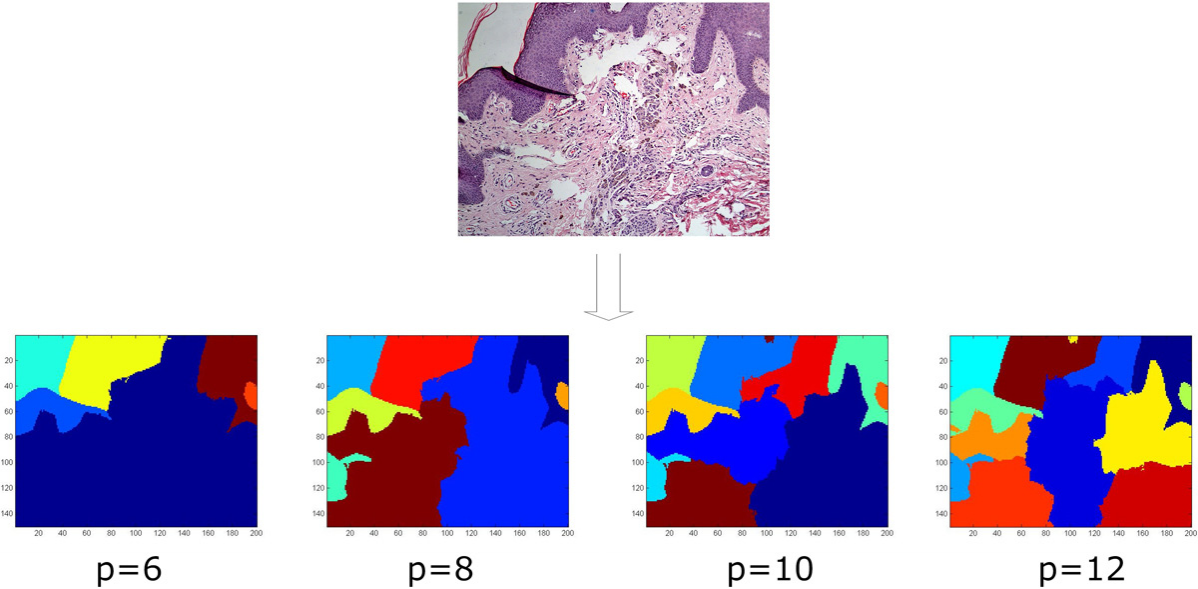
**NCut outputs of different parameter setting**. Running NCut with *p *set to 6, 8, 10 and 12. By increasing *p*, more and more inner structures can be detected. The parameter *p *controls the recognition level of the model.

The number of regions *p *is a parameter to be set beforehand. Figure 4 shows the NCut outputs of the same image with different parameter settings. Parameter *p *will affect the model performance to some extent. We will present this in the discussion section.

#### Feature extraction based on 2D-DWT

Previous work on skin image analysis has indicated that a good feature extraction method significantly affect model performance. Many problem-oriented feature expression methods have been proposed and proven to be successful in histopathology and dermatopathology [1]. However, feature extraction methods for skin biopsy images are seldom reported. Considering the variation of colour, rotation, magnification and even resolution in skin biopsy images, we propose a transformation-invariant feature extraction method based on 2-dimension discrete wavelet transformation (2D-DWT). The basic idea of the proposed feature extraction originated from [20,17], which suggested applying 2D-DWT in colour space for each block within a given image. We briefly describe the proposed feature extraction methods as follow.

**Figure 5:**
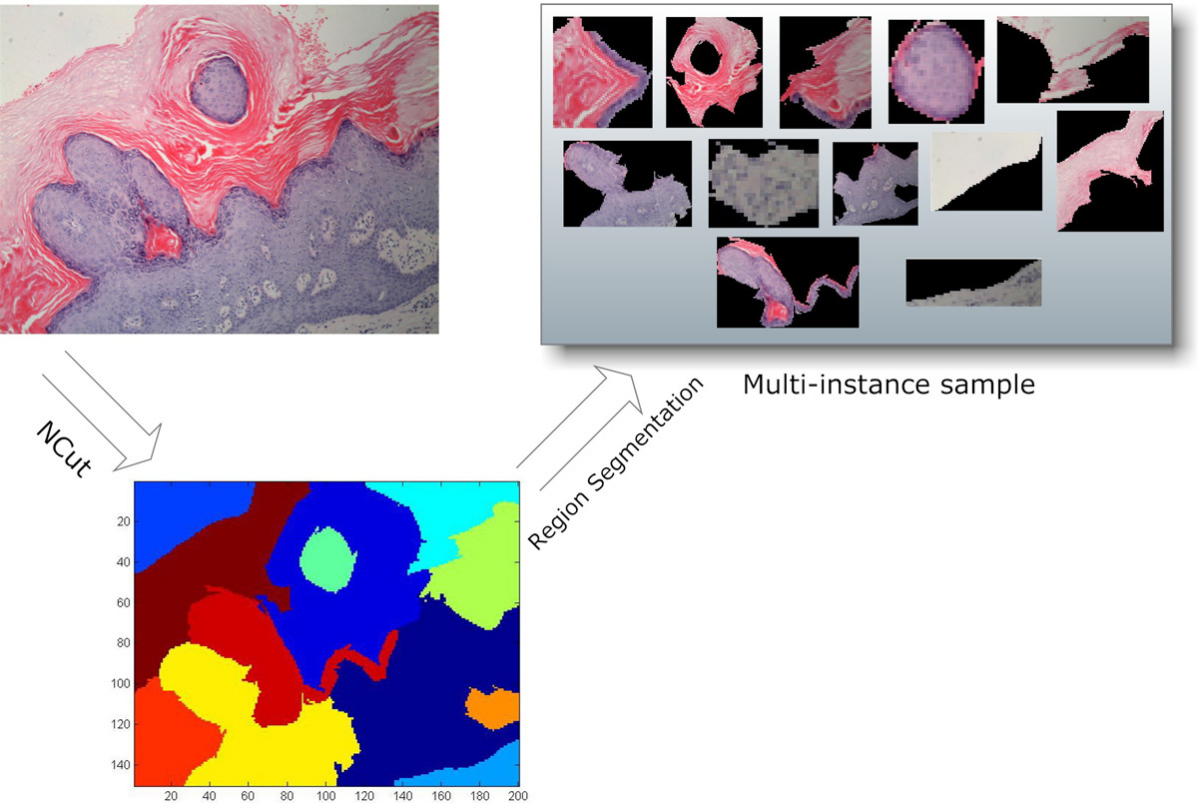
**Minimum covering rectangle of NCut output**. An example of generating the minimum covering rectangle of NCut outputs. For convenient expression and processing, we store the pixels of the minimum bounding rectangle that exactly covers an NCut generated irregular region. Only minimum rectangles whose edges are parallel to the edges of the original image are stored, rather than the optimal rectangle that could be drawn by rotating the image to any angle.

1. Input a local region *IR *generated by NCut. Note that regions generated by NCut are irregular. For convenience, we store them as minimum covering rectangles by padding the regions with black pixels, as indicated in Figure 5. This padding does not significantly affect model performance, as most of these padding pixels will be discarded in later steps.

2. Colour space transformation. *IR *is an RGB expression and now transferred to LUV space, denoted as *IR_LUV*. Calculate features *f*_1 _= *mean*(*IR_LUV.L*), *f*_2 _= *mean*(*IR_LUV.U*) and *f*_3 _= *mean*(*IR_LUV.V*).

3. Divide *IR_LUV *into squares of size *m × m *pixels, resulting in (*width/m*) *× *(*height/m*) blocks, denoted as *Bpq*, where *p *= {1, ..., *width/m*} and *q *= {1, ..., *height/m*}. Eliminate blocks that are totally black, so as to remove padding pixels as much as possible.

4. Apply 2D-DWT to each *B_pq_*, and keep coefficients LH, HL and HH. Let $t_{x}=\sqrt{\frac{1}{4}x^{T}x)}$, where *x *∈ {*LH*, *HL*, *HH*}. Average *t_x _*for all blocks within a region to obtain features *f*_4_, *f*_5_, f_6_.

5. Following [20], calculate the normalized inertia of order 1, 2 and 3 as features *f*_7_, *f*_8_, *f*_9_.

After the above 5 steps, a 9-ary real vector is obtained for each region. An image is transformed into a set of disjoint regions, represented as real feature vectors. Thus we turn the original dataset into a multi-instance representation. Note that this representation is invariant to transformation, as 2D-DWT extracts texture features of regions that are irrelevant to rotation angle and magnification. The other features, LUV mean and normalized inertia of orders 1, 2 and 3, are also transformation-invariant. In the following section, we will provide an in-depth discussion of the effectiveness of this feature extraction method.

#### Feature extraction based on SIFT

Scale-invariant feature transform (SIFT) [21] is a well-studied feature extraction method widely used in the study of medical image classification. Juan C. Caicedo et al. [10] used SIFT to extract histopathological image features. We apply SIFT as our second feature extraction strategy. Unlike 2D-DWT, SIFT has been proven to be a robust key point selector in different image annotation and analysis applications. We use the common setting of SIFT, in which 8 orientations and 4 × 4 blocks are used, resulting in a 128-ary vectorial expression. Intuitively speaking, SIFT selects several outstanding points to represent a given image. We apply SIFT to the NCut-generated regions to obtain a features vector.

### Model training

We propose two multi-instance learning algorithms to train our model. The first algorithm is based on Citation-KNN [22], and the second is a Bayesian multi-instance learning algorithm, namely Gaussian Process Multi-Instance Learning (GPMIL) [23]. Citation-KNN was first proposed by Jun Wang et al. [22] and can be regarded as a multi-instance version of traditional KNN classifiers. To determine a given test bag's label, Citation-KNN considers not only the *K *nearest labelled bags, i.e., references, but also labelled bags that regard the given bag as a *K *nearest neighbour, i.e., citers. Citation-KNN is well studied and has many successful applications in machine learning. GPMIL introduced a Gaussian process prior and solved the multi-instance learning problem in a Bayesian learning framework. The essential idea of GPMIL is that by defining a set of latent variables and the likelihood function, it establishes the relationship between class labels and instances in a probabilistic framework. By imposing a Gaussian process prior on these latent variables, we can use a Bayesian learning strategy to derive a posterior distribution of annotation terms given a training dataset and a test image.

We extend these two algorithms to meet the requirements of our annotation task, taking into consideration some insights into skin biopsy image annotation. On the one hand, because there is no prior knowledge on which to base multi-instance learning assumptions [24] for our task, we build model from the original assumption [15]. Citation-KNN with a properly defined similarity metric is a simple but effective algorithm in this case. On the other hand, the confidence level of a term to be annotated to a given image is preferred, which requires us to model the predictive distribution of annotation terms. To achieve this goal, we extend Bayesian learning to the multi-instance setting and model the posterior distribution of the annotation terms. An additional benefit of the Bayesian learning framework is that it is possible to model correlation between annotation terms, leading to a more general model.

#### Citation-KNN for annotation

Citation-KNN is a multi-instance learning algorithm inspired by the citation and reference system in scientific literature. To determine the label of a test bag *X*, it considers not only the neighbours (references) of *X *but also the bags (citers) that regard *X *as a neighbour. Citation-KNN uses both references and citers to determine an unseen bag's concept label. The key problem is how to evaluate distances between bags to identify references and citers.

Citation-KNN implements a simple idea: that if two images *A *and *B *share with the same term, they should regard each other as neighbors under a properly defined similarity measure, i.e., *B *is one of the *K *nearest neighbors of *A *and vice versa. In our work, a modified version of Hausdorff distance [25] was used as a similarity measure, which is given by

(3) $$AHD\left(A,B\right)=\frac{\underset{a\in A}{\sum }\underset{b\in B}{\text{min}}d\left(a,b\right)+\underset{b\in B}{\sum }\underset{a\in A}{\text{min}}d\left(b,a\right)}{\left|A|+|B\right|}$$

where *AHD *measures the average Hausdorff distance between two bags *A *and *B*, and *a, b *are instances in each bag. *d*(*x*, *y*) is the Euclidean distance function in instance space. As indicated in [25], *AHD *achieves a better performance than other set distance functions in multi-instance learning. The intuitive definition of *AHD *is the average minimal distance between instances from two bags, which better evaluates the spatial relationship between a pair of bags.

Note that Citation-KNN is a memory-based algorithm, meaning that all training samples must be stored when testing a given image and that no training procedure is required. When testing, *AHD *must be computed between the test image and all training samples. To reduce the computation cost, we define a locality matrix *LM *to speed up the algorithm as follow.

1. Cluster the training set *D *to obtain *s *clusters and denote the centroid of each cluster as *c_i_*, *s *= {*i *= 1, ..., *s*}.

2. Compute the *AHD *distance between each training sample and each centroid *s_i_*, and keep the *K *nearest training samples for each *s_i _*in the *ith *row of *LM*.

Thus we obtain a *s*-by-*K *locality matrix *LM*. When testing an image, we first calculate the distance between centroids and the given image, then discard the centroids that are far from the given image. For the remaining centroids, we perform a table lookup on *LM *to find the corresponding rows of the remaining centroids; only the training samples associated with such rows are needed in distance computation. We can prune out a large portion of the training samples that are far away from the test image, which greatly reduces the computational cost. The matrix can be computed only once before testing with cost *O*(*n*^2^), where *n *= *|D| *stands for the size of the training set.

#### GPMIL

We propose a Bayesian learning algorithm with a Gaussian process prior for our annotation task. Following [23], we first introduce an unobserved latent function *g*(*x*) defined in instance space for each annotation term *t *such that for each instance *x*, *g*(*x*) gives a probability indicating the confidence of *x *to be annotated with term *t*. We further impose a Gaussian process prior on all *g*(*x*) of the whole instance space. Let *G *= {*g*(*x_i_*)*|i *= 1, ..., *n_inst_*}, where *n_inst _*denotes the size of the instance space. We have *G ~ N *(0, *K*) as a Gaussian process prior [26], where *K *is a Gram matrix of some well-known kernel of all instance pairs. To establish the connection between *g*(*x*) and the annotated terms of images, a likelihood function is defined according to the basic multi-instance assumption [15] as Eq. (4):

(4) $$p\left(t|G_{B}\right)=p(t|g(x_{1}),\cdots \;,g(x_{\left|B\right|})=\underset{j}{\text{max}}(g(x_{j}))$$

where *G_B _*represents the output of *g*(*x*) for all instances in bag *B*, and *|B| *is the size of bag *B*. For mathematical convenience, *softmax *is used instead of *max*, thus we have

(5) $$p\left(t|G_{B}\right)=\underset{j}{\text{max}}\left(g\left(x_{j}\right)\right)\approx 1\text{n}\underset{x\in B}{\sum }e^{\alpha g\left(x\right)}$$

where *α *is an amplifying factor of the *softmax *function. If the largest *g*(*x_j_*) for any *j *is less than 0.5, bag *B *would not be annotated with term *t *because *p*(*t|G_B_*) *<*0.5. The joint likelihood function on the whole training set *D *can be written as

(6) $$p\left(T|G_{D}\right)=\underset{B\in D}{\prod }p\left(t|G_{B}\right)$$

where *T *is a boolean vector indicating whether each bag *B *in *D *is annotated with term *t*. However, we are concerned with the label of a test bag *B*, not *GB *or *GD *themselves. Following Bayes rule, the posterior distribution over *G *for training dataset *D *and term *t *can be written as:

(7) $$p\left(G_{D}|D,T\right)=\frac{p\left(T|G_{D}\right)p\left(G_{D}\right)}{p\left(T|D\right)}$$

where *p*(*T|G_D_*) is the joint likelihood defined in Eq. (6), *p*(*G_D_*) is the Gaussian process prior and *p*(*T|D*) is the marginal likelihood given by

(8) $$p\left(T|D\right)=\int p\left(T|G_{D}\right)p\left(G_{D}\right)dG_{D}$$

With Eq. (7) and (8), we can further obtain the prediction distribution of a test bag *X *for annotating term *t *as

(9) $$p\left(t|D,T,X\right)=\int p\left(t|G_{X},X\right)p\left(G_{X}|D,T,X\right)dG_{X}$$

where in the right hand side of Eq. (9), *p*(*t|G_X_*, *X*) represents the likelihood function of the test mage *X*, given by $p\left(t|G_{X},X\right)=\int p\left(G_{X}|G_{D},D,X\right)p\left(G_{D}|D,Y\right)dG_{D}$, and *p*(*G_X_|D*, *T*, *X*) represents the posterior distribution of latent variable *G_X_*. For each test image *X*, using the whole training dataset and the corresponding annotation vector *T*, we can obtain a predictive distribution that is a function of *X *and *t*. The effective method for solving Eq. (9) can be found in [27,23].

To make the idea of GPMIL clearer, we provide an example as follows:

1. Suppose we have a training image set *D *associated with a binary annotation vector for term *t *and a test image *X*.

2. Following Eq. (4) and (6), calculate the likelihood function for the training set *D*.

3. Following Eqs. (7), (8) and (9), we write down the analytical form of the predictive distribution for *X*.

4. We use some approximate method to transform the predictive distribution to a Gaussian distribution that can be solved analytically. After this step, a close-form solution can be obtained for testing any unseen images. In other words, the training set can be discarded in the testing step.

For each annotation term *t*, a model is trained by using GPMIL. For a test image, each model calculates a probability indicating the confidence of annotating the image with the corresponding term.

## Evaluation

### Dataset description

We evaluated the proposed method using a real skin biopsy image dataset from The Second Affiliated Hospital of Guangzhou University of Chinese Medicine and The Third Affiliated Hospital of SUN YAT-SEN University.. The dataset contains diagnosis data from 2010 to 2012, including 2734 patient records and 6691 skin biopsy images associated with a set of standard dermatopathology annotations in Chinese. The dataset was generated by manually selecting 2-3 biopsy images at the same magnification ratio for each patient. Each term indicates a certain feature of concern in the biopsy images of a certain patient. Each image has pixels with 24k colours in RGB space with a size of 2048 × 1536 pixels. We considered 15 annotation terms in the evaluation, among which some often appear in lesion regions and others are only observed for some special types of skin diseases. Table 1 lists these terms with their rates of occurance in the evaluation dataset.

**Table 1:** 15 annotation terms with occurence rates

**No**.	Name	Rate
t1	hyperkeratosis	28.65%
t2	parakeratosis	22.71%
t3	absent granular cell layer	1.8%
t4	acanthosis	32.15%
t5	thin prickle cell layer	4.14%
t6	hyperpigmentation of Basal cell layer	6.48%
t7	Munro microabscess	2.61%
t8	nevocytic nests	9.12%
t9	infiltration of lymphocytes	36.99%
t10	basal cell liquefaction degeneration	4.46%
t11	horn cyst	6.31%
t12	hypergranulosis	8.25%
t13	follicular plug	3.72%
t14	papillomatosis	16.48%
t15	retraction space	4.53%

A binary matrix is obtained by text matching, in which each row is a 15-ary binary vector indicating whether an image has been annotated with these terms. Based on domain knowledge, a skin biopsy image is possibly composed of up to 15 regions. We set the number of regions *p *as 8, 10 or 12 for separate runs of our proposed algorithm, then combine them through majority voting. Images fed to NCut are all rescaled to 200 × 150 pixels for effective calculation. The feature extraction methods were applied to the rescaled images instead of the original ones because the rescaled images contain sufficient information.

### Evaluation criteria

As mentioned in the previous section, we decomposed the annotation task into several binary classification tasks. *Zero-one loss *(also called *precision*) is a straightforward criterion for our task. Because multiple terms are associated with an image, multi-label machine learning evaluation criteria are also suitable for our task. We also introduce *Hamming loss *for evaluation, whose definition can be found in [28]. Intuitively speaking, *Hamming loss *is a measure of how many object-term pairs are annotated by mistake. Note that larger values of *Hamming loss *indicate better model performance. *Zero-one loss *evaluates the annotation performance of a single term, whereas *Hamming loss *evaluates the whole model output for all terms.

### Evaluation results

#### Evaluation of feature extraction and model training methods

We evaluated the performance of two feature extraction methods 2D-DWT, and SIFT, combined with two model training algorithms. The purpose was to show the effectiveness using different feature expressions to different models. We used the following configuration. The whole dataset was randomly divided into a training set and a testing set with a ratio 3:7. The number of regions generated by NCut was set to 10. The block size for 2D-DWT was set to 4 × 4. Images were all rescaled to 200 × 150 for effective computation. SIFT was used with its default settings, as mentioned above. For GPMIL, because the model provides a probability *r*, it can be converted into a binary value through *b *= *sign*(*r −*0.5). We also implemented the bag-of-features method with an RBF kernel function [10] as a baseline for comparison. For every model, we ran 10 trials and averaged all of the results to obtain a final result. Table 2 shows the results, measured by *zero-one loss*, for the annotation of 15 terms.

**Table 2:** Precisions of different models

Term	Citation		GPMIL		BOF
	2D-DWT	SIFT	2D-DWT	SIFT	

t1	63.24%	59.06%	**65.14%**	64.55%	58.05%
t2	66.45%	67.12%	67.56%	**67.63%**	64.34%
t3	69.54%	66.47%	**70.40%**	68.29%	57.93%
t4	73.88%	70.85%	**77.78%**	72.23%	74.55%
t5	59.12%	60.21%	**62.12%**	58.23%	56.71%
t6	63.41%	63.00%	65.12%	**66.02%**	58.55%
t7	69.42%	71.23%	71.98%	70.24%	**76.60%**
t8	70.04%	66.73%	**73.12%**	69.44%	62.86%
t9	78.19%	79.11%	75.00%	**81.49%**	76.82%
t10	**72.42%**	68.48%	71.34%	69.49%	64.03%
t11	81.42%	80.91%	85.12%	83.23%	**81.95%**
t12	75.00%	74.83%	73.52%	**78.56%**	74.82%
t13	80.12%	78.02%	**83.13%**	80.04%	77.85%
t14	**84.21%**	82.35%	82.34%	83.12%	80.48%
t15	81.23%	80.34%	83.55%	**85.90%**	79.22%

In Table 2 the column BOF stands for the result of the bag-of-features method proposed in [10]. The best result in each row has been highlighted in bold. It can be observed that the multi-instance learning-based methods are superior to the bag-of-features-based method for annotating most terms. Both feature extraction methods achieved the best performance in some cases. We cannot simply determine which method is superior to the other. Some prior knowledge or experience can be introduced to determine the most suitable feature representation method. Another factor that should be noted is the stability of the proposed method, which achieves higher precision but lower variance compared to the baseline method, meaning that the proposed method is more reliable and stable for the annotation of different terms.

Table 3 illustrates the performance as evaluated by *Hamming loss*. GPMIL with 2D-DWT feature representation achieves the best *Hamming loss*. Note that *Hamming loss *is often higher than the average error rate for the annotation of all terms, as the correct annotations may not be in the same image, leading to some increase in *Hamming loss*.

**Table 3:** Hamming loss of different models

Citation		GPMIL		BOF
2D-DWT	SIFT	2D-DWT	SIFT	
31.24%	29.56%	**26.54%**	27.02%	35.03%

#### The impact of number of regions

**Figure 6:**
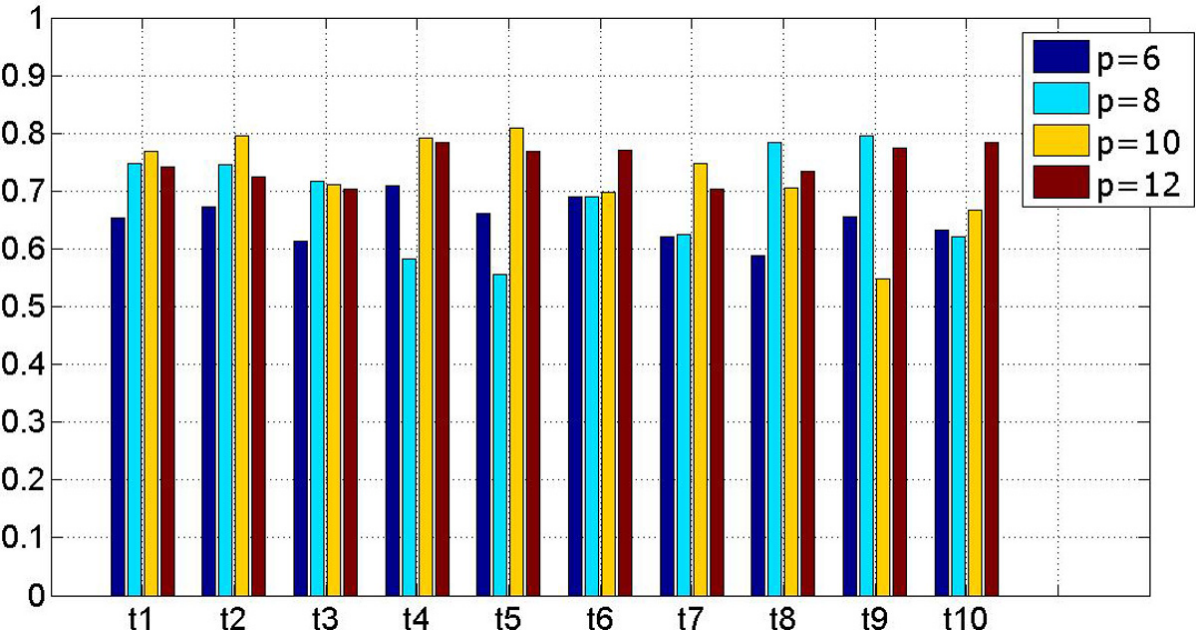
**Evaluation results of different numbers of regions**. The model performance evaluated by *precision *with different *p*. A large value of *p *means small simple regions, each of which corresponds to just one or two terms.

We varied the number of regions generated by NCut to demonstrate its impact on the model performance and reveal the relationship between the proposed method and clinical experience. We used 2D-DWT as the only feature extraction method and varied *p *from 6 to 12 in step 2. As indicated in Figure 4, a small *p *value may lead to complex regions featured as more terms, whereas a large *p *value may lead to fragments of regions. Figure 6 shows the results for the first 8 terms.

We can see that the parameter *p *affects model performance to some extent. In most cases, it is true that a larger *p *means better performance. Even in cases where *p *= 6, the proposed algorithm achieves an acceptable result while annotating some terms, which is in opposition to our experience, as we do not know which number of regions would be best. We propose to use an ensemble method to create a model with better generalisation, reducing the impact of an improper setting of *p*. To do this, we adopted a majority voting strategy; a model is trained with each value of *p *when testing an image, and the models vote to determine the final result. Because the models are of binary outputs, they vote for each annotation term. Table 4 shows the ensemble result for each term.

**Table 4:** Ensemble results of different numbers of regions

Citation		GPMIL		BOF
2D-DWT	SIFT	2D-DWT	SIFT	
31.24%	29.56%	**26.54%**	27.02%	35.03%

#### The impact of an imbalanced training set

**Figure 7:**
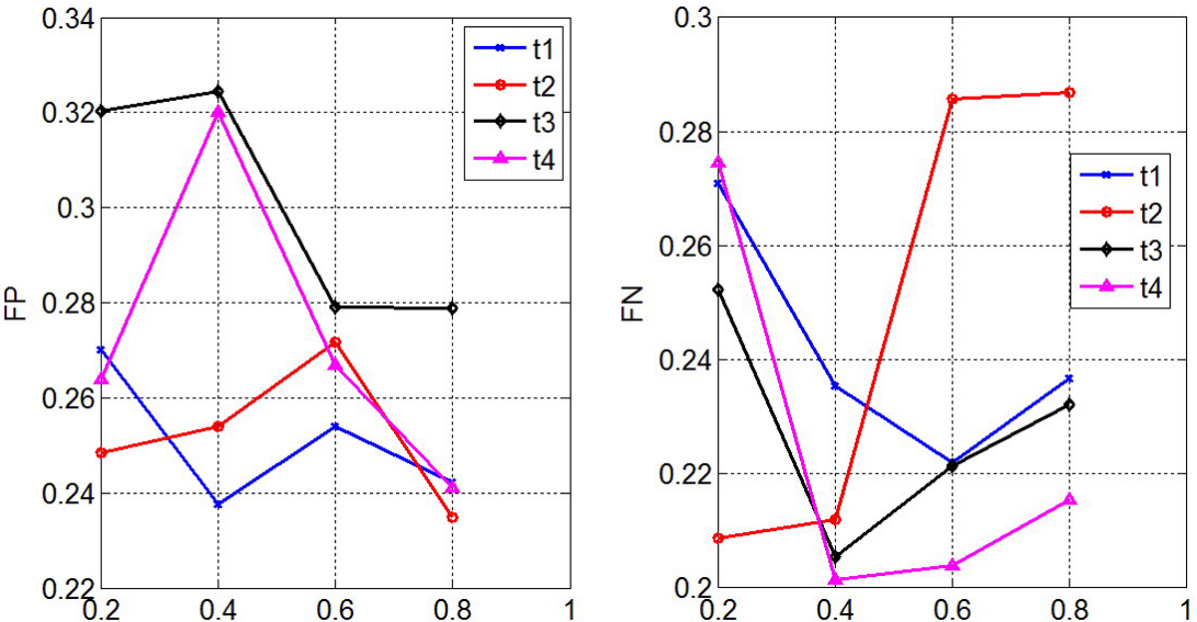
**The impact of an imbalance of the training dataset**. Evaluation results on training datasets of different imbalance levels by changing the ratio between positive and negative samples. The impact of dataset imbalance was evaluated based on the false positive (FP) and false negative (FN) rates.

As indicated in Table 1 the frequency of different terms varies significantly. When training a model with an imbalanced dataset, the model would be biased toward the major class. We varied the ratio *r *between positive and negative samples to determine a good strategy for building a training dataset. To do this, a series of datasets *Dr *of size *N *are constructed by first randomly selecting *N *× *r *images annotated with a term from the training set, then randomly selecting *N *× (1 - *r*) images not annotated with the same term. We used Citation KNN and 2D-DWT feature extraction for this evaluation. Note that in this case accuracy may not be a proper measure because the model tends to predict all test samples as one class when training with a highly imbalanced dataset. For example, when a dataset is composed of 90% positive and 10% negative samples, a model that always makes positive predictions would achieve an accuracy of 90%. However, this accuracy would be meaningless. We used false positive (FP) and false negative (FN) ratios to measure accuracy. Figure 7 shows the model performance of different values of *r *for the first 4 terms.

#### An illustration of the model output

**Figure 8:**
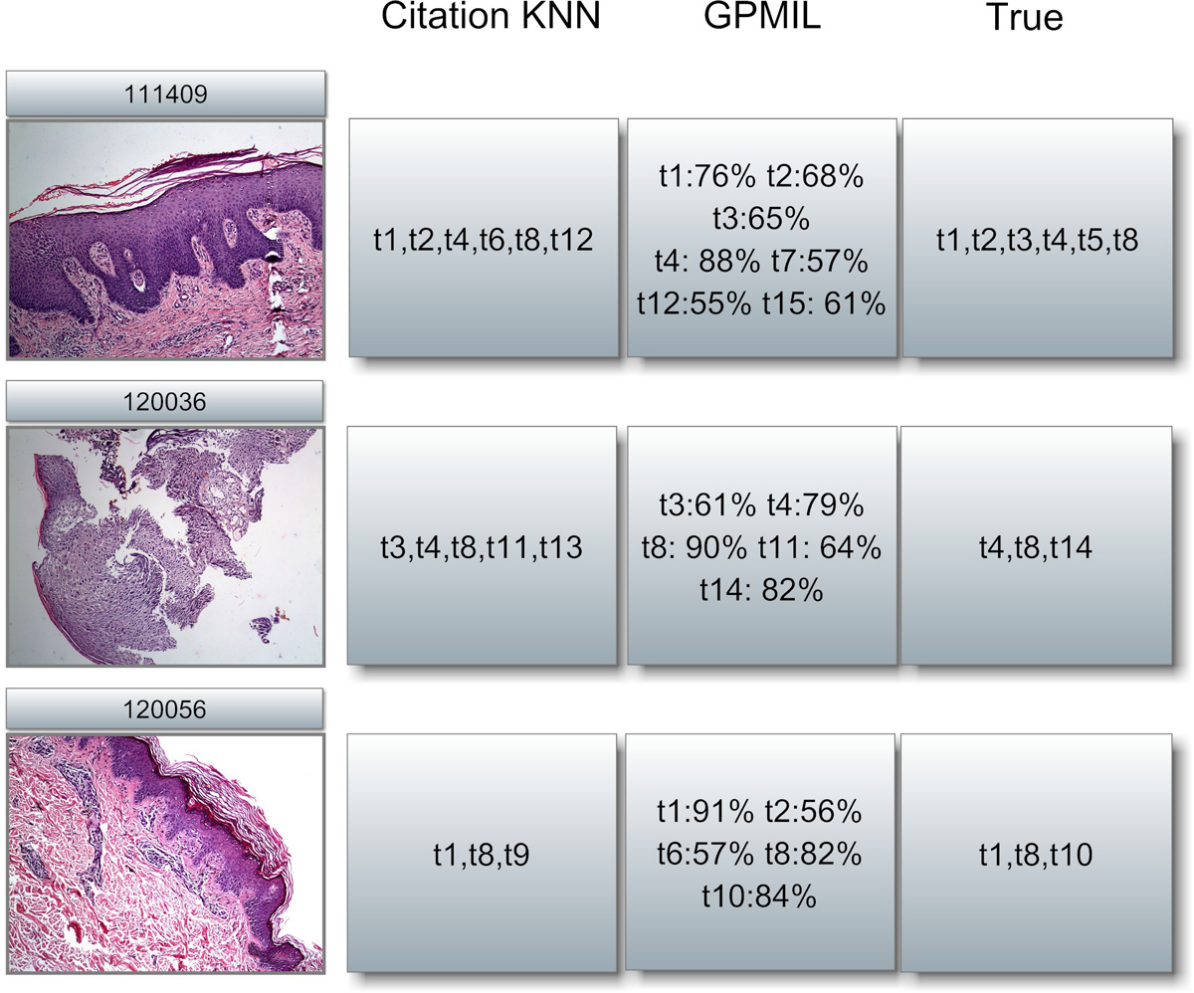
**An illustration of actual outputs**. An illustration of actual output of the proposed models. Three skin biopsy images were manually selected from the dataset and then applied to the two proposed models. For Citation KNN, a set of terms were obtained. For GPMIL, a set of terms with probabilities were obtained indicating the confidence of the model. We omitted terms with a probability of less than 50%.

Finally, we illustrated a comparison between the model output and the real annotation terms attached to the test images. We selected three images from the evaluation dataset. The three images were taken in 2011 from three different patients. Figure 8 illustrates the annotation results of Citation-KNN and GPMIL. The column **True **stands for annotation terms that belong to the images according to the diagnosis records. Citation KNN provides a set of terms and GPMIL further outputs a confidence level for the terms. In Figure 8, we omitted terms with a probability of less than 50%.

## Discussion

### Multi-instance representation vs. bag-of-features

In histopathological and dermatopathological image analysis, a large amount of work was based on bag-of-features construction [10,29-31], in which a dictionary is built whose elements are small patches from a set of training images and can be regarded as keywords. To classify or annotate a given image, these methods need only examine the presence or quantity of keywords in the image. Thus the image can be expressed as a histogram of elements in the dictionary.

Our multi-instance framework is quite different from bag-of-features-based methods. The proposed framework retains original features through direct feature extraction methods, whereas bag-of-features-based methods only generate some statistical measures, e.g., histogram of the elements in a dictionary, which may cause some loss of discriminative information. Meanwhile, the elements of a dictionary in a bag-of-features-based method are often derived from grid-based image patches. We argue that such patches are not able to fully capture the essential discriminative information contained in histopathological images. The proposed framework generates meaningful local regions with visually disjoint edges using NCut, which is more consistent with diagnostic experience in dermatopathology.

### Number of regions of Normalized Cut

We addressed some issues related to setting a reasonable number of regions. Though the evaluation results showed that an ensemble with different regions yields an acceptable result, this method lacks a good explanation. When inspecting skin biopsy images, a small number of regions indicates that the doctor is focusing on relatively global features, whereas a large number indicates more detailed features. Doctors' behaviour may range from global to detailed according to their knowledge and experience. Skin tissue is composed of three anatomically distinct layers, namely the epidermis, dermis, and subcutaneous tissue (fat). Epidermis can be further divided into four layers. Each layer has a distinctive stained colour and special structures. Distinct pathological changes involving any of these whole layers such as Hyperkeratosis, Acanthosis and Hyperpigmentation of the basal cell layer, can be easily recognised in a small number of segmentations. Specific changes within a layer, such as a Munro microabscess, nevocytic nests or infiltration of lymphocytes, can be more accurately detected when the image is divided into more pieces. Either a global or a detailed view is reasonable in diagnosis, which is consistent with the above evaluation results.

### Relationship between regions

Considering the relationships between regions, it should be noted that skin tissues have clearly featured inner structures. Some correlation can be observed between the presence of different terms within an image. For example, terms such as hyperkeratosis and parakeratosis can only be found in certain regions and above features such as acanthosis or hyperpigmentation of the basal cell layer (if the term is attached to the same image). Theoretically speaking, GPMIL can capture such correlations to some extent by defining a different likelihood function [27]. Our Gaussian process prior for GPMIL also implies such relationships. However, previous work [32] reported that the inclusion of such relationships did not make a positive contribution to model performance. We owe this phenomenon to the doctors' experience implied in the training dataset, i.e., that doctors or experts pay more attention to important local regions, which statistically reduces the emphasis on relationships between regions.

## Conclusion

In this work, we introduce the application of multi-instance representation and learning to the recognisation and annotation of dermatopathological skin biopsy images. To reprensent a skin biopsy image as a multi-instance sample, we apply Normalized Cut to divide an image into visually disjoint regions and then extract features for each region through 2D-DWT and SIFT-based algorithms. Two training algorithms have been proposed for model building: Citation KNN provides a binary output, and GPMIL calculates a probability indicating the confidence level of the model output. The evaluation results show that the proposed method is effective for biopsy image recognition and annotation.

Medically, the results contribute to the development of dermatopathology. Time-consumption and expenditure would be lower if a computer program could take over the annotation work of a pathologist. The accuracy of diagnosis would be increased if subjective factors, such as a doctor's skill, and objective factors, such as light, were eliminated. The application accords with developing trends in dermatopathology. Further work will include introducing relationships between terms in multi-instance multi-label framework and designing more powerful region recognition and feature extraction methods.

## Competing interests

The authors declare that they have no competing interests.

## Authors' contributions

Gang Zhang and Ziping Li are equally contributing authors of this article. Gang Zhang was responsible for the main framework of this article, as well as algorithm development and programming, and he wrote the first draft of the paper. Ziping Li was responsible for study design and crude data processing, and he revised the paper from a clinical perspective. Honglai Zhang is the coordinator who organised the study, Xiangyang Shu provided useful advice on dermatology, and Jian Yin and Guozheng Li provided useful advice on data processing.

## Declarations

The publication costs for this article were funded by the corresponding author.

This article has been published as part of *BMC Medical Genomics *Volume 6 Supplement 3, 2013: Selected articles from the IEEE International Conference on Bioinformatics and Biomedicine 2012: Medical Genomics. The full contents of the supplement are available online at http://www.biomedcentral.com/bmcmedgenomics/supplements/6/S3.
